# An integrated strategy for efficient vector construction and multi-gene expression in *Plasmodium falciparum*

**DOI:** 10.1186/1475-2875-12-373

**Published:** 2013-10-26

**Authors:** Jeffrey C Wagner, Stephen J Goldfless, Suresh M Ganesan, Marcus CS Lee, David A Fidock, Jacquin C Niles

**Affiliations:** 1 Department of Biological Engineering, Massachusetts Institute of Technology, Cambridge, MA 02139, USA; 2 Department of Microbiology and Immunology, Columbia University College of Physicians and Surgeons, New York, NY 10032, USA; 3 Division of Infectious Diseases, Department of Medicine, Columbia University College of Physicians and Surgeons, New York, NY 10032, USA

## Abstract

**Background:**

The construction of plasmid vectors for transgene expression in the malaria parasite, *Plasmodium falciparum*, presents major technical hurdles. Traditional molecular cloning by restriction and ligation often yields deletions and re-arrangements when assembling low-complexity (A + T)-rich parasite DNA. Furthermore, the use of large 5′- and 3′- untranslated regions of DNA sequence (UTRs) to drive transgene transcription limits the number of expression cassettes that can be incorporated into plasmid vectors.

**Methods:**

To address these challenges, two high fidelity cloning strategies, namely yeast homologous recombination and the Gibson assembly method, were evaluated for constructing *P. falciparum* vectors. Additionally, some general rules for reliably using the viral 2A-like peptide to express multiple proteins from a single expression cassette while preserving their proper trafficking to various subcellular compartments were assessed.

**Results:**

Yeast homologous recombination and Gibson assembly were found to be effective strategies for successfully constructing *P. falciparum* plasmid vectors. Using these cloning methods, a validated family of expression vectors that provide a flexible starting point for user-specific applications was created. These vectors are also compatible with traditional cloning by restriction and ligation, and contain useful combinations of commonly used features for enhancing plasmid segregation and site-specific integration in *P. falciparum*. Additionally, application of a 2A-like peptide for the synthesis of multiple proteins from a single expression cassette, and some rules for combinatorially directing proteins to discrete subcellular compartments were established.

**Conclusions:**

A set of freely available, sequence-verified and functionally validated parts that offer greater flexibility for constructing *P. falciparum* vectors having expanded expression capacity is provided.

## Background

Malaria continues to be a leading cause of morbidity and mortality worldwide. Nearly 50% of the global population is at risk, and in 2010 there were an estimated 219 million cases and 660,000 deaths [1]. *Plasmodium falciparum* is the parasite pathogen responsible for the most virulent disease. No vaccine is clinically approved to prevent malaria. Treatment relies heavily on the use of a limited number of anti-malarial drugs to which resistance is increasingly widespread [2], which makes it critical to identify new and effective drugs. Using genetic approaches to validate potential drug targets in *P. falciparum* is pivotal to this effort. However, the process of constructing the plasmid vectors needed for these studies is time-consuming and inefficient, and imposes a significant barrier to genetically manipulating the parasite.

Several aspects of parasite biology interact to create this challenge. First, the parasite’s genome is extremely (A + T)-rich (80-90%) [3], and extended regions of low complexity sequence are common [4,5]. Second, regulatory 5' and 3' UTR sequences are poorly defined in *P. falciparum*, and large regions of putative regulatory DNA are needed to facilitate robust transgene expression [6]. Very few 5′ and 3′ UTRs have been precisely mapped. As a result, 1-2 kb 5′ and 3′ UTRs are frequently selected on the assumption that these comprise the information necessary to support efficient transcription [6-9]. These long UTRs are close to 90% in (A + T) composition. Third, the mean coding sequence (CDS) length in *P. falciparum* (excluding introns) is 2.3 kb, nearly twice that of many model organisms [3].

Gene complementation is a powerful strategy, used extensively in forward genetics studies in other organisms, but this approach is under-utilized in *P. falciparum* due in large part to the challenges associated with efficiently assembling the necessary complementing constructs [10]. The ability to more routinely construct expression vectors for complementation studies is highly synergistic with the increasing rate at which genome-wide insertional mutagenesis studies are identifying candidate genes associated with growth, cell cycle and other phenotypic defects in *P. falciparum*[11,12]. In constructing over-expression, complementation and gene targeting vectors in *P. falciparum*, long (A + T)-rich regions must be cloned into final plasmids that can exceed 10 kb. It is recognized that the traditional and commonly used restriction/ligation-based cloning method is inefficient for assembling these vectors, and often yields plasmids with regions that are deleted and/or re-arranged [13]. Consequently, time-consuming screening of large numbers of bacterial clones is needed to increase the probability of recovering the intact target vector, if it is at all present.

In addition to the vector assembly challenges, typical over-expression vectors are limited in the number of transgenes that can be simultaneously expressed. In the most common format, two expression cassettes are available, and one of these is dedicated to expressing a selectable marker [7]. Increasing the expression capacity of a single plasmid can be accomplished by introducing additional 5'UTR-CDS-3'UTR cassettes, but this further complicates vector construction for reasons described above. This problem has been circumvented in several eukaryotes through the use of a viral 2A-like peptide that prevents peptide bond formation between two specific and adjacent amino acids during translation and results in the production of two separate proteins from a single expression cassette [14]. Recently, the 2A signal has been shown to be functional in *P. falciparum*[15], but its broader utility with respect to proteins that are trafficked to different subcellular parasite compartments has not been examined.

Here, an inexpensive and straightforward strategy for more robustly and flexibly assembling *P. falciparum* vectors is introduced, while simultaneously maximizing the amount of transgenic information expressible from a single plasmid without using additional 5′UTR-CDS-3′UTR expression cassettes. This has been achieved by developing a family of vectors that integrate use of high fidelity and robust DNA assembly by yeast homologous recombination [16] and *in vitro* assembly by the isothermal chew-back-anneal Gibson method [17] with traditional restriction/ligation-based cloning. Additionally, several desirable utility features have been consolidated in this vector family, including: site-specific integration mediated by the *Bxb1* integrase [18]; improved plasmid segregation mediated by either Rep20 elements [19] or a *P. falciparum* mini-centromere (*pfcen5-1.5*) [20]; and all of the currently used *P. falciparum* selection markers. Lastly, the broader utility of a viral 2A-like peptide to achieve expression from a single cassette of multiple genes targeted to distinct parasite subcellular compartments has been demonstrated. This resource is freely available through the Malaria Research and Reference Reagent Resource Center (MR4) [21].

## Methods

### Molecular biology

Unless otherwise indicated, enzymes were from New England Biolabs (Ipswich, MA, USA) and chemicals were from Sigma-Aldrich (St. Louis, MO, USA) or Research Products International (Mt. Prospect, IL, USA). High fidelity (HF) restriction enzymes were used when available. PCR was routinely performed with Phusion DNA polymerase in HF Buffer, or with a 15:1 (v:v) mixture of Hemo KlenTaq:Pfu Turbo (Agilent, Santa Clara, CA, USA) in Hemo KlenTaq Buffer. The latter conditions permit PCR amplification directly from parasite culture samples, usually included at 5% of the total reaction volume. Plasmids were prepared for transfection with maxi columns (Epoch Life Science, Missouri City, TX, USA) or the Xtra Midi Kit (Clontech, Mountain View, CA, USA).

### Vector construction

The primers used for these studies are listed in Additional file 1.

### Yeast homologous recombination

Yeast homologous recombination (HR) vector construction was carried out by standard methods [16,22]. Variable amounts of vector backbone (typically 0.1-2 μg) were digested using standard methods to generate linearized vector. PCR was carried out using standard techniques to generate fragments for insertion bearing 20-40 bp homology to the desired flanking regions on the vector. Competent *Saccharomyces cerevisiae* W303-1B was prepared as described [23] and frozen at -80°C. Either unpurified or column-purified PCR product was co-transformed with either unpurified or column-purified linearized vector. A wide range of concentrations of both linearized vector and PCR product were observed to be efficacious. Transformed yeast were plated on YPD agar (10 g/L yeast extract, 20 g/L peptone, 20 g/L dextrose, 20 g/L agar) supplemented with 400 mg/L G-418 disulphate and allowed to grow for 48-72 hours at 30°C. Typical yields were 10-100 colonies for a negative control transformation lacking PCR insert, and 50-1,000 colonies for the complete HR reaction.

Colonies were then either harvested by plate scraping or picked and grown overnight in YPD + 400 μg/mL G-418. Cells were then treated with 2 U zymolyase (Zymo Research, Irvine, CA, USA) in 250 μL buffer ZB (10 mM sodium citrate pH 6.5, 1 M sorbitol, 25 mM EDTA and 40 mM dithiothreitol) for 1 hour at 37°C to generate spheroplasts. Yeast spheroplasts were lysed with the addition of 250 μL buffer MX2 (0.2 M NaOH and 10 g/L sodium dodecyl sulphate). Plasmid DNA was then purified either by spin column (Epoch Life Science, Missouri City, TX, USA) or alcohol precipitation.

The recovered DNA was transformed directly into *Escherichia coli* DH5α or EPI300 (Epicentre Biotechnologies, Madison, WI, USA) prepared with a Z-Competent Transformation Kit (Zymo Research) or transformed by electroporation. Occasionally, the DNA mixture was drop dialyzed against water for 20 min prior to transformation to increase electroporation. Plasmid DNA was isolated from colonies and assayed for correct vector assembly by restriction digest, diagnostic PCR and/or DNA sequencing.

### Gibson assembly

Isothermal chew-back-anneal assembly, commonly known as Gibson assembly, was carried out as described [17]. Briefly, vector and PCR product were prepared in the same way as for yeast HR assembly. Fragments were combined with either a home-made or commercially available Gibson Assembly Master Mix. The home-made Master Mix was prepared by combining 699 μL water, 320 μL of 5× isothermal reaction buffer (500 mM Tris-Cl, pH 7.5, 250 mg/mL PEG-8000, 50 mM MgCl_2_, 50 mM dithiothreitol, 1 mM each of four dNTPs, 5 mM NAD), 0.64 μL T5 Exonuclease (Epicentre, 10 U/μL), 20 μL Phusion DNA polymerase (2 U/μL) and 160 μL Taq DNA ligase (40 U/μL). This solution was divided into 20 μL aliquots and stored at -20°C. Generally, >100 ng of linearized vector was added to the mixture with an equal volume of PCR insert, generating a variable vector: insert ratio. The mixture was incubated at 50°C for 1 hour and 0.5 μL was transformed into *E. coli* as described above.

### Restriction/ligation cloning

Restriction/ligation cloning was carried out by standard techniques. Ligations were incubated overnight at 16°C and heat inactivated prior to transformation.

### Construction of *attP*-containing (pfYC3 series) plasmids

The 2 × *attP* fragment was PCR amplified from pLN-ENR-GFP [18] with primers SG702/703. pfYC120:FL and pfYC140:FL were digested with SalI and combined with gel-purified PCR product in a Gibson assembly reaction to obtain pfYC220:FL and pfYC240:FL, respectively. These vectors were then digested with MluI and PmlI to release the fragment containing the Rep20 and CEN/ARS elements. Approximately 100 ng of digested vector was then combined in a Gibson assembly reaction containing 50 nM each SG814 and SG815 to recircularize the vector while adding a unique PmeI site between the MluI and PmlI sites. This yielded pfYC320:FL and pfYC340:FL. For integration at the *cg6* locus, these two vectors were co-transfected with pINT [18] (~50 μg each) into *P. falciparum* 3D7-*attB* (MRA-845 from MR4 [21]). Clonal populations were obtained by limiting dilution and integration verified by PCR using the SG864/865 primer pair.

### Construction of the *pfcen5-1.5* mini-centromere containing (pfYC4 series) plasmids

The *pfcen5-1.5* element was amplified from *P. falciparum* 3D7 genomic DNA with primers SG894/SG928. pfYC102:FL and pfYC104:FL were digested with MluI and PmlI and the gel-purified backbone lacking the 2×*Rep20* element was attached to the SG894/SG928 PCR product by Gibson assembly to yield pfYC402:FL and pfYC404:FL, respectively. Clones were verified by restriction digest and by sequencing with SG369.

### Cloning *Plasmodium falciparum ama1* and *trxR* genes into pfYC120

The *ama1* and *trxR* genes were amplified from *P. falciparum* 3D7 genomic DNA using the AMA1 pcDT F/R and Trx pcDT F/R primer pairs, respectively. Restriction/ligation, Gibson assembly and yeast HR were performed as described above.

### Multi-cistronic constructs using the T2A for evaluating subcellular trafficking rules

After Western blot and microscopic imaging analysis, the identity of each strain was re-verified by PCR amplifying and sequencing a uniquely identifying fragment of the transfected construct using the SG763/764 and SG502/646 primer pairs, respectively.

### Parasite culture and transfection

*Plasmodium falciparum* strain 3D7 parasites were cultured under 5% O_2_ and 5% CO_2_ in RPMI-1640 media supplemented with 5 g/L Albumax II (Life Technologies), 2 g/L NaHCO_3_, 25 mM HEPES-K pH 7.4, 1 mM hypoxanthine and 50 mg/L gentamicin. Transfections used ~50 μg of each plasmid and were performed by the spontaneous DNA uptake method [24] or by direct electroporation of ring-stage cultures [25]. Transgenic parasites were selected with 2.5 mg/L Blasticidin S, 1.5 μM DSM-1, 5 nM WR9920 (Jacobus Pharmaceuticals) and/or 250 mg/L G-418 beginning 2-4 days after transfection.

### Monitoring transfection progress by luciferase expression

Firefly and *Renilla* luciferase levels were measured every fourth day after transfection using the Dual-Luciferase Reporter Assay System (Promega). Samples for measurement were prepared by centrifugation of 1.25 mL of parasite culture at 2% haematocrit to generate an ~25 μL parasite-infected RBC pellet. Pellets were either used immediately or stored at -80°C until needed for luciferase measurements.

### Parasite DNA extraction and qPCR analysis

DNA was harvested from schizont stage parasites at ≥ 5% parasitaemia. Infected red blood cells (RBCs) were treated with 0.1 mg/mL saponin in PBS to release parasites, which were either immediately used or stored in liquid nitrogen for later analysis. Parasites were lysed for 1 hour at 50°C in 200 μL of 50 mM Tris-Cl pH 8.0, 50 mM EDTA, 0.5 mg/mL SDS and 10 μL of protease solution (Qiagen). After adding RNase A (28 U; Qiagen), reactions were incubated at 37°C for 5 min. After adding 20 μL of 6 M sodium perchlorate, DNA was isolated by phenol/chloroform extraction and ethanol precipitation.

qPCR reactions (20 μL each) contained 1× Thermopol Buffer, 0.2 mM dNTPs, 200 nM relevant primer pair (Additional file 2), 0.5× SYBR Green I (Life Technologies), 0.1 μL Taq DNA polymerase and 5 μL of a DNA dilution. Thermocycling was performed on a Roche LightCycler 480 II for 40 cycles according to the following programme: 95°C for 20 sec; denature: 95°C for 3 sec; anneal/extend: 60°C for 30 sec; fluorescence measurement. Vector-borne amplicons (drug resistance marker genes) and a native chromosomal locus (β-actin) were quantified by comparison with plasmid or PCR-amplified DNA standards, respectively.

### Western blot

Approximately 10^6^ late-stage parasites were harvested by lysis of infected RBCs with 0.5 g/L saponin and then lysed by heating in urea sample buffer (40 mM dithiothreitol, 6.4 M urea, 80 mM glycylglycine, 16 g/L SDS, 40 mM Tris-Cl, pH 6.8) at 95°C for 10 min. After separation by SDS-PAGE, proteins were transferred to a PVDF membrane and probed with an antibody against firefly luciferase (FL) (Promega G7451), neomycin phosphotransferase II (Millipore 06-747) or green fluorescent protein (Abcam ab1218). Blots were then imaged using a horseradish peroxidase-coupled secondary antibody and SuperSignal West Femto substrate (Thermo Scientific).

### Northern blot

Total RNA was purified from infected RBCs with a combination of Tri Reagent RT Blood (Molecular Research Center) and an RNeasy Mini Kit (Qiagen). One mL of parasite culture at 20% haematocrit and ~10% late-stage parasitaemia was frozen on liquid nitrogen and thawed with the addition of 3 mL Tri Reagent RT Blood. After phase separation with 0.2 mL BAN (Molecular Research Center), 2 mL of the upper aqueous phase was mixed with 2 mL ethanol and applied to an RNeasy Mini column for purification according to the manufacturer’s instructions. Total RNA (6.5 μg) for each sample (with or without the addition of 6 pg FL RNA generated by *in vitro* transcription) was mixed with an equal volume of denaturing sample buffer (95% formamide, 0.25 g/L SDS, 0.25 g/L bromophenol blue, 0.25 g/L xylene cyanol, 2.5 g/L ethidium bromide, 50 mM EDTA) and heated at 75°C for 10 min before loading on a 1% TAE agarose gel. Electrophoresis was performed at 80 V for 75 min and RNA was transferred to a Nylon membrane (Pall Biodyne Plus) by downward capillary transfer in 50 mM NaOH for 90 min. After UV fixation (Stratagene Stratalinker, 1.25 mJ), the membrane was probed and imaged with the North2South Chemiluminescent Detection Kit (Thermo). The biotinylated FL probe was prepared from a DNA template generated by PCR with primers SG311 and SG313.

### Fluorescence microscopy

For live cell imaging, parasite cultures were incubated for 20 min with 30 nM MitoTracker Deep Red FM (Life Technologies). Infected RBCs were then washed with phosphate-buffered saline and applied to poly-L-lysine-coated glass-bottom culture dishes (MatTek, Ashland, MA, USA). Attached cells were overlaid with RPMI media (free of phenol red and Albumax II) containing 2 μg/mL Hoechst 33342 (Sigma), and imaged immediately at room temperature using a Nikon Ti-E inverted microscope with a 100× objective and a Photometrics CoolSNAP HQ2 CCD camera. Images were collected with the Nikon NIS Elements software and processed using ImageJ [26].

## Results

### Vector family design and features

In creating this plasmid vector resource several useful design criteria have been incorporated, namely: (1) access to multiple, orthogonal and high-fidelity strategies for cloning a target fragment into the identical context; (2) pre-installed utility features including access to all commonly used *P. falciparum* selection markers (*bsd*^*1*^, *hdhfr*, *ydhodh* and *nptII*), plasmid integration sequences (*attP* sites) [18], and plasmid segregation/maintenance features such as Rep20 [19] and the mini-centromere, *pfcen5-1.5*[20]; (3) sufficient modularity to permit straightforward tailoring for user-specific needs; and, (4) ease of manipulation using reagents that are readily prepared in-house or commercially available at low cost.

**Figure 1:**
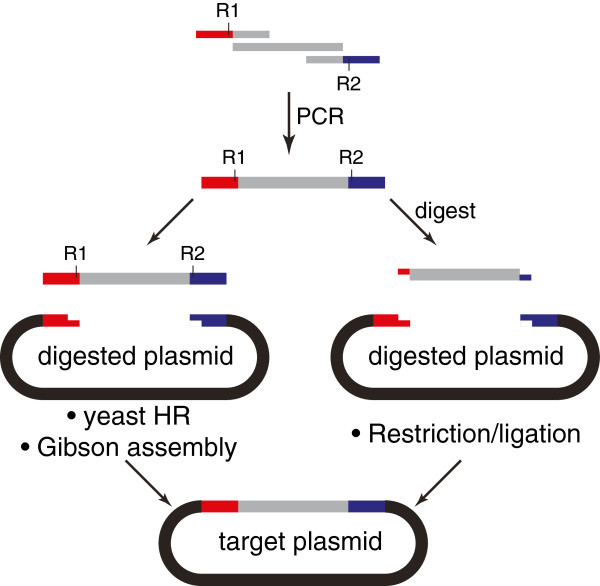
**Schematic of the homology-based (yeast HR and Gibson assembly) and traditional restriction/ligation cloning strategies selected as part of an integrated framework for the orthogonal assembly of *Plasmodium falciparum *constructs.** Beginning with a common primer set, PCR products and the desired vector backbone (see Figure 2 for details), the identical target plasmid can be assembled using any of these approaches individually or in parallel.

This vector family framework includes access to yeast homologous recombination (HR) [16], Gibson assembly [17] and restriction/ligation as central cloning strategies (Figure 1). The challenges associated with the traditionally used restriction/ligation method when cloning *P. falciparum* sequences have been described [13]. It is thought that the observed genomic deletions and re-arrangements are related to the long (A + T)-rich regions in combination with the restriction and ligation process and the instability of these constructs in *E. coli*. Though inefficient overall, this strategy is used successfully, and so it is preserved as an option that interfaces directly with the more efficient yeast HR and Gibson strategies.

A major advantage of both Gibson and yeast HR strategies over traditional restriction ligation based cloning is that they do not require enzymatic digestion of the inserted fragment, which can impose constraints on cloning target DNA that contains these restriction sites internally. Rather, as they depend on homologous ends overlapping with a digested vector, the insert does not need to be digested. This allows greater flexibility by permitting a larger set of restriction sites on the vector to be used. Yeast HR requires more overall time compared to Gibson and restriction/ligation cloning, as *S. cerevisiae* grows more slowly than *E. coli*. However, this strategy efficiently yields target constructs and all the key components can be inexpensively generated in-house [23]. The Gateway® strategy (Life Technologies) has also been used to construct *P. falciparum* vectors [27]. This approach has not been included in the current study, as it is significantly more expensive than the methods described here. However, when needed, the features required for enabling Gateway® cloning should be straightforward to introduce into the framework described below.

**Figure 2:**
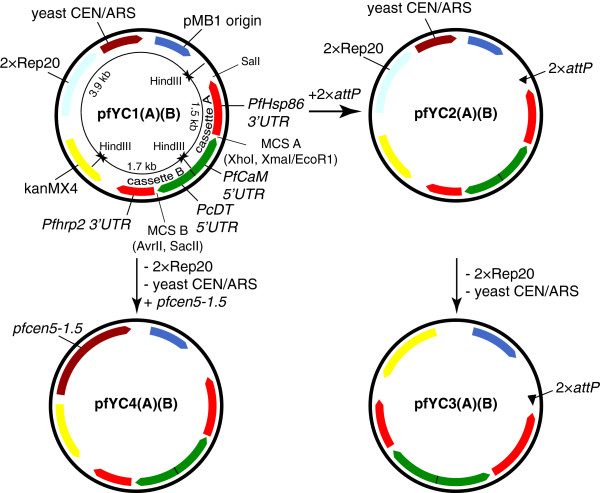
**Schematic summary of the new family of plasmid vectors.** Plasmids are designated by the pfYC prefix, a series number (1-4) and a number (0-4) defining the resistance marker present in expression cassette A (5′*PfCaM*/3′*PfHsp86* UTRs) or B (5′-*PcDT*/3′*PfHRPII* UTRs). The series number is defined by specific utility features included in the plasmid as follows: 1 = yeast CEN/ARS origin to enable plasmid maintenance in *S. cerevisiae* during yeast HR and a 2 × Rep20 element to improve plasmid segregation in *P. falciparum*[19]; 2 = same as in 1, but with a 2 × *attP* element added to enable site-specific chromosomal integration into existing *attB*^*+*^ strains [18]; 3 = 2 × *attP* element is present, but the yeast CEN/ARS origin and 2 × Rep20 elements have been eliminated; and 4 = the *pfcen5-1.5* mini-centromere element is included to facilitate plasmid segregation and maintenance at single copy in *P. falciparum*[20], while the yeast origin, 2 × Rep20 and 2 × *attP* elements have been eliminated. *P. falciparum* resistance markers are designated as: 0 = none; 1 = *nptII* (G-418 resistance); 2 = *bsd* (Blasticidin S resistance); 3 = *hdhfr* (WR99210 resistance); and 4 = *ydhodh* (DSM-1 resistance). A non-resistance gene cloned into the available expression cassette is indicated by a colon followed by the gene name (e g, pfYC110:FL indicates that the *nptII* and firefly luciferase genes are present in expression cassettes A and B, respectively). Three HindIII sites present on the base plasmid are noted, as they are useful for topologically mapping these vectors and derivatives to screen for potential rearrangements and large insertions or deletions.

The overall architecture of this new vector family and the built-in utility features are summarized in Figure 2, and is derived from the pfGNr plasmid previously deposited as MRA-462 in MR4. This plasmid contains bacterial (pMB1) and yeast (CEN6/ARS4) origins of replication, and the *kanMX4* gene under the control of a hybrid bacterial/yeast promoter to facilitate selection of bacterial or yeast colonies on kanamycin or G-418, respectively. This plasmid contains two *P. falciparum* gene expression cassettes consisting of the commonly used 5′/3′UTR pairs *PfCaM*/*Pfhsp86* and *PcDT*/*PfHRPII* arranged head-to-head to improve transcriptional efficiency [28]. In *P. falciparum*, plasmid selection using G-418 is enabled by a *gfp-nptII* gene fusion expressed from the *PcDT/PfHRPII* cassette, and a 2×Rep20 element to enhance plasmid segregation during replication is also present [19].

From this vector, a library of eight base plasmids was first created in which each of the four frequently used *P. falciparum* selection markers was cloned into one of the two *P. falciparum* expression cassettes. For ease of reference, a nomenclature to describe the various vector family members was defined. Plasmids are designated as pfYC*xAB*, where *x* is a series number indicating the presence of a specific set of utility features (1 = Rep20/yCEN, 2 = Rep20/yCEN/2×*attP*, 3 = 2 × *attP* and 4 = *pfcen5-1.5*) and *A* and *B* denote the resistance marker expressed from the *PfCaM/PfHsp86* (cassette A) and *PcDT*/*PfHRPII* (cassette B) UTR pairs, respectively (0 = no marker; 1 = *nptII*; 2 = *bsd*; 3 = *hdhfr*; and 4 = *ydhodh*). Introducing the 2 × *attP* site, which facilitates site-specific integration mediated by the *Bxb1* integrase into compatible *attB* strains [18], at the SalI site yields the pfYC2 plasmid series. Two representative members, namely pfYC220:FL and pfYC240:FL (Additional file 3), were generated in this study and provide a standardized approach for easily generating the entire set. Both the pfYC1 and pfYC2 plasmids facilitate manipulation through yeast homologous recombination, Gibson assembly and traditional restriction/ligation cloning to provide the greatest flexibility in assembling a specific construct.

A limited set of pfYC3 (pfYC320:FL and pfYC340:FL) plasmids have also been generated, and these retain the *attP* site but not the Rep20 and CEN6/ARS4 elements from the pfYC2 plasmid series. Elimination of the Rep20 and CEN6/ARS4 elements from plasmids intended for integration into the *P. falciparum* genome may be desirable, as the Rep20 element has the potential to induce transcriptional silencing in a subtelomeric chromosomal context [29]. Likewise, the *S. cerevisiae-*derived CEN6/ARS4 element could possibly behave aberrantly when integrated into a *P. falciparum* chromosome. A limited set of pfYC4 plasmids (pfYC402:FL and pfYC404:FL) has been made in which the Rep20 and CEN6/ARS4 elements in the pfYC1 series have been replaced by the mini-centromere *pfcen5-1.5*. The option to use yeast homologous recombination in the pfYC3 and pfYC4 series is eliminated. However, Gibson assembly and/or traditional restriction/ligation can be used to generate final constructs that are immediately ready for integration. Validated procedures for generating the complete set as dictated by user needs have also been provided.

### Vector construction using various cloning methods

**Figure 3:**
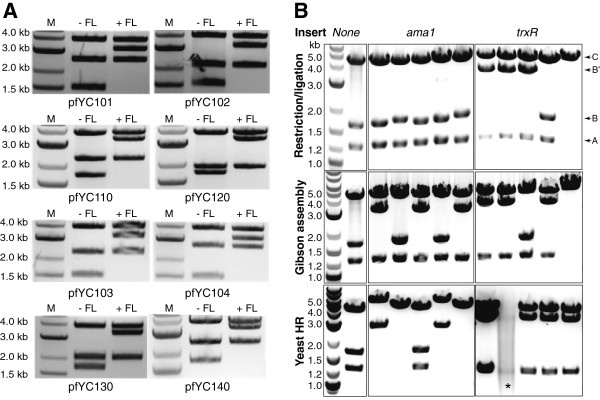
**Heterologous and native *Plasmodium falciparum *genes can be successfully assembled into pfYC vectors using all three cloning strategies. (A)** The firefly luciferase gene (*FL* = 1.65 kb) was cloned into the pfYC1 and pfYC3 series (Additional file 3) using either yeast HR or Gibson assembly. Topological mapping with HindIII digestion yields three fragments, as *FL* and the selection markers do not contain HindIII sites. A 3.9 kb fragment is released from the pfYC1 series whether *FL* is present or not (Figure 2). The fragments containing cassettes A and B from pfYC10x:*FL* plasmids are (1.5 kb + *FL*) = 3.2 kb and (1.7 + selection marker size) kb, respectively. Similarly, the fragments containing cassettes A and B from pfYC1x0:*FL* plasmids are (1.5 + selection marker size) kb and (1.7 + *FL* size) = 3.4 kb, respectively. The sizes of the different selection markers are: *nptII* (0.8 kb); *hdhfr* (0.6 kb); *bsd* (0.4 kb) and *ydhodh* (0.95 kb). **(B)** Two native *P. falciparum* genes, *ama1* (apical membrane antigen 1; PF3D7_1133400; 1.87 kb) and *trxR* (thioredoxin reductase; PF3D7_0923800.1; 1.85 kb) were cloned in parallel using restriction/ligation, Gibson assembly and yeast HR, and the same PCR products and digested pfYC120 vector. Successful gene insertion is expected to yield three HindIII digestion products that include: a backbone fragment (denoted as C); cassette B with the *ama1* or *trxR* gene inserted (denoted as B when no insert is present and B′ when containing the proper insert); and cassette A containing the *bsd* gene (denoted as A). As a reference, the parent pfYC120 plasmid yields products denoted as A, C and B upon HindIII digestion. The asterisk in the yeast HR *trxR* panel denotes sample degradation that occurred during storage prior to analysis by gel electrophoresis.

Several vectors were constructed to illustrate the ability to successfully clone firefly and *Renilla* luciferase reporter genes, and two native *P. falciparum* genes (*ama1* and *trxR* both ~1.85 kb and ~70% in (A + T) content) into this vector family using all three cloning strategies. Using yeast HR or Gibson assembly, firefly or *Renilla* luciferase was cloned into the available expression cassette of the entire pfYC1 series (Additional file 3). All vectors were sequenced and topologically mapped by HindIII restriction digestion. As shown in Figure 3A, final plasmids with the expected topology can be assembled using these methods. Similarly, the candidate *P. falciparum* genes *ama1* and *trxR* were inserted into pfYC120 using the three vector assembly methods in parallel. Cloning reactions were carried out using the same insert and vector preparations to minimize differences between the materials used in each reaction. Five colonies derived from each cloning method were screened for each gene target and mapped by HindIII digestion to establish proper assembly of the target vector (Figure 3B). Gibson assembly yielded topologically correct plasmids for both gene targets. However, under the conditions tested, yeast HR and restriction/ligation yielded the expected plasmid for *trxR* only. Overall, these data show that all three methods can be used to successfully clone native *P. falciparum* genes into this new vector family. Importantly, these independent cloning strategies allow use of the same plasmid backbone and insert combinations to assemble the identical final construct, thus improving the flexibility and overall ease with which *P. falciparum* vectors are made.

### Plasmids in this vector family can be maintained as stable episomes and chromosomally integrated in *Plasmodium falciparum*

**Figure 4:**
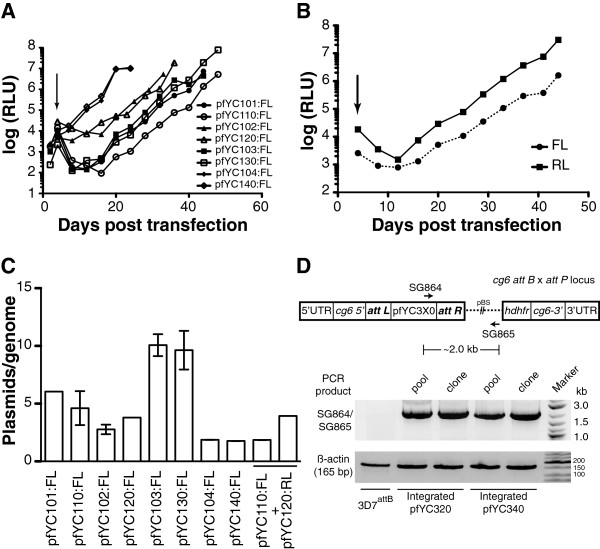
**The pfYC plasmid family exhibits typical behaviour during *Plasmodium falciparum *transfection, and can be maintained episomally and chromosomally integrated. (A and B)** The entire pfYC1xx:FL plasmid series was either transfected individually **(A)** or as a single pair (pfYC110:FL + pfYC120:RL) **(B)** under the appropriate drug selection initiated on day 4 post-transfection (arrow). Firefly and *Renilla* luciferase levels were monitored to assess parasite population growth kinetics until a parasitaemia ≥1% was attained. **(C)**. The copy number of each plasmid per parasite genome was determined for both the single and double transfections. **(D)** PCR confirmation of chromosomal integration of pfYC320 and pfYC340 at the *cg6* locus in the *P. falciparum* 3D7-attB strain. The β-actin gene was PCR amplified as a positive control.

Toward establishing this vector family as a verified resource and a framework for routine use in *P. falciparum* transgenic experiments, their ability to yield stable episomal and integrated *P. falciparum* lines was evaluated. The entire pfYC1AB:FL vector set was transfected either singly or in a paired combination (pfYC110/pfYC120) into *P. falciparum* strain 3D7. Transfected parasites were selected using the appropriate drug(s), and growth was monitored by following luciferase activity. As shown in Figure 4A, parasites transfected with these plasmids were successfully selected with typical kinetics [30,31]. Interestingly, under the conditions tested, the pfYC104:FL- and pfYC140:FL- transfected parasites selected with DSM-1 emerged more rapidly than parasites selected with Blasticidin S, WR99210 or G-418. Dual plasmid transfected parasites emerged at rates similar to those observed in single plasmid transfections (Figure 4B). Copy numbers for the various pfYC1 plasmids were also determined by quantitative PCR using the single-copy chromosomal β-actin gene as a reference. These data indicate that plasmids selected with Blasticidin S, DSM-1 and G-418 are maintained at an average ≤ five copies, and for WR99210 at ~ ten copies per parasite genome (Figure 4C). This is consistent with results using other *P. falciparum* vectors [7,20], indicating that the pfYC vector family behaves similarly to currently used plasmids and is suitable for use in transgenic experiments.

Frequently, the ability to site-specifically integrate constructs is preferred to ensure stable, homogeneous transgene expression at single copy. The pfYC3 plasmid series is designed to accomplish this by combining cloning strategy flexibility and a site-specific integration *attP* utility feature [18], while eliminating plasmid elements that are potentially deleterious when chromosomally integrated (Rep20 and CEN6/ARS4). As validation of this desired behaviour, 3D7-*attB* parasites were transfected with pfYC320:FL and pfYC340:FL. Stable parasite lines expressing FL were selected under Blasticidin S or DSM-1 pressure, respectively, and site-specific integration at the *cg6* locus was detected by PCR both at the population level and in isolated clones (Figure 4D). Overall, these data collectively show that the pfYC vector family provides a robust and complementary set of high efficiency and timesaving cloning strategies for enabling routine assembly of DNA constructs that can be successfully used in *P. falciparum* transgenic experiments. Of note, while we have assembled two representative pfYC4 series plasmids containing the *pfcen5-1.5* centromere element as a useful starting point for future use, we have not evaluated these in transfections.

### Expanded transgene expression from a single plasmid that is compatible with proper subcellular trafficking

**Figure 5:**
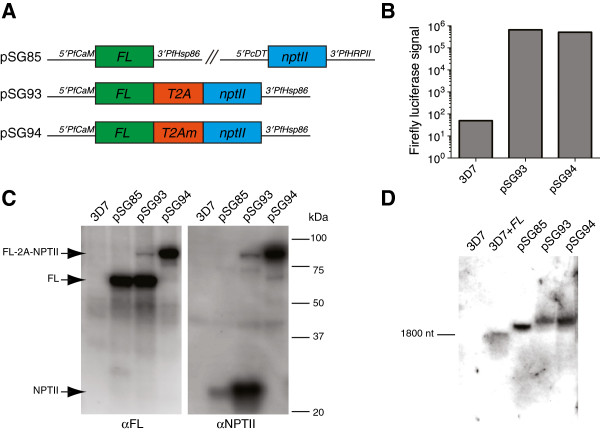
**The *Thosea asigna *virus 2A-like peptide (T2A) enables expression of two functional proteins in *Plasmodium falciparum *from a single expression cassette*. *(A)** Schematic of *FL-nptII* and control constructs. **(B)** Both T2A- and T2Am- containing constructs produce active FL. **(C)** Western blot detection of FL- and nptII- containing proteins. **(D)** Northern blot analysis of *FL-*containing transcripts in transfected parasites. 3D7 + *FL* indicates the inclusion of a synthetic *FL* mRNA produced by *in vitro* transcription.

The ability to simultaneously express multiple proteins from a single plasmid irrespective of their subcellular localization can be highly useful, as it reduces the need for sequential transfections and limits exhausting the small set of available selection markers. The virus-derived 2A-like peptide sequences (2A tags), which have been used successfully in mammalian, yeast, plant and protozoan contexts to enable polycistronic expression from a single eukaryotic mRNA [14,15] were used to accomplish this. These 2A tags mediate peptide bond “skipping” between conserved glycine and proline residues, yielding one protein with a short C-terminal extension encoded by the tag, and the other with an N-terminal proline. The small size (eight conserved amino acid positions) and broad cross-species functionality of the 2A tag makes it an attractive candidate for application to *P. falciparum,* an organism in which this technology has not been extensively explored. As an entire expression cassette is usually committed exclusively to expressing a selection marker, an initial experiment was designed to address whether the *Thosea asigna* virus 2A-like sequence (T2A) could be used to expand the number of genes expressed from this cassette without compromising the ability to select transfected parasites. T2A with a short, N-terminal linker region [32] was inserted between the FL and *nptII* genes in cassette A to generate pfYC101:FL-2A-nptII. A control construct containing a non-functional tag (T2Am), in which two conserved residues are mutated to alanine [32] was also generated (Figure 5A). These plasmids were transfected into *P. falciparum* 3D7 under G-418 selection pressure and obtained resistant parasites with FL activity (Figure 5B), demonstrating the production of functional nptII and FL proteins in both cases. The ability of T2A to produce distinct FL and nptII proteins from a single mRNA was confirmed by Western and Northern blot (Figure 5C and 5D, respectively). As expected, mutating T2A to T2Am eliminates the formation of discrete proteins, but does not alter the size of the *FL-nptII* mRNA. This initial characterization, in addition to demonstrating T2A functionality in *P. falciparum,* highlights the potential for using T2A to recover valuable expression capacity by encoding additional information into existing selection marker cassettes while eliminating the unpredictability of how a protein fusion will function.

**Figure 6:**
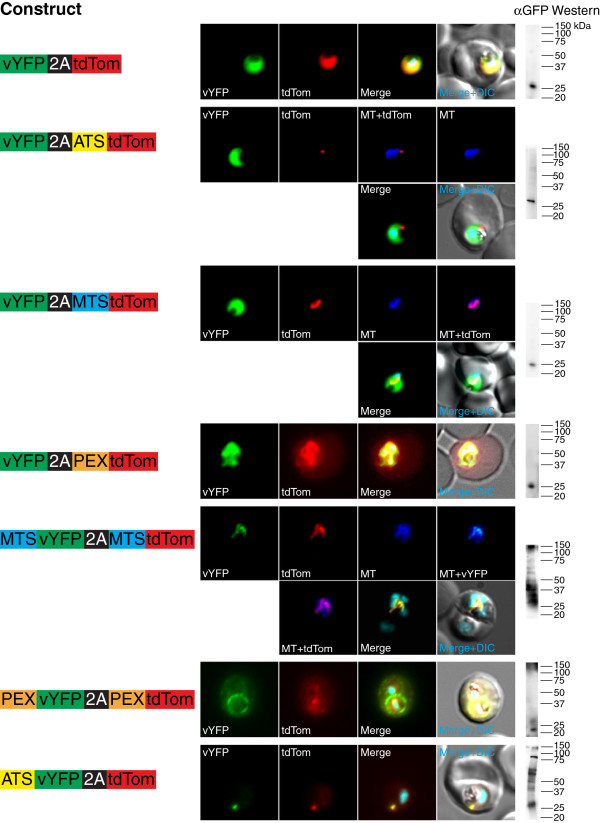
**The 2A sequence can be used to successfully and predictably target proteins to distinct subcellular compartments.** Various targeting sequences were N-terminally fused to an upstream vYFP and a downstream tdTom reporter separated by T2A. The vYFP and tdTom proteins were localized using direct fluorescence microscopy imaging. Mitochondria were stained with MitoTracker (MT), and nuclei with Hoechst 33342. Legend: ATS = apicoplast targeting sequence; MTS = mitochondrial targeting sequence and PEX = protein export element.

Next, the flexibility with which T2A can be used to produce dicistronic messages encoding proteins destined for distinct subcellular compartments within the parasite and its RBC host was examined. Several dicistronic constructs encoding an N-terminal Venus yellow fluorescent protein (vYFP) and a C-terminal tdTomato protein (tdTom) separated by T2A were built in the pfYC120 vector. Previously validated apicoplast, mitochondrial and RBC export targeting sequences derived, respectively, from: acyl carrier protein (PF13_0208500; aa 1-60 = ATS) [33], HSP60 (PF13_1015600; aa 1-68 = MTS) [34], and knob-associated histidine-rich protein (PF13_0202000; aa 1-69 = PEX) [35] were used. Seven contexts were created in which a different protein targeting signal (or none at all) was placed immediately upstream of vYFP and/or tdTom as follows: (a) vYFP-2A-tdTom; (b) vYFP-2A-ATS-tdTom; (c) vYFP-2A-MTS-tdTom; (d) vYFP-2A-PEX-tdTom; (e) MTS-vYFP-2A-MTS-tdTom; (f) PEX-vYFP-2A-PEX-tdTom; and, (g) ATS-vYFP-2A-tdTom. vYFP and tdTom trafficking were evaluated by fluorescence imaging microscopy, and production of vYFP *versus* a possible fusion to tdTom was distinguished by Western blot (Figure 6). Overall, when no targeting sequence was upstream of vYFP, the downstream tdTom was faithfully trafficked to the subcellular compartment based on the associated targeting sequence. Similarly, when vYFP and tdTom are associated with the same targeting sequence (parasite cytosol, mitochondrion and RBC cytosol tested), both were trafficked as separate proteins to the same subcellular compartment, as expected. For the ATS-vYFP-2A-tdTom construct, vYFP was trafficked to the apicoplast as expected. Interestingly, a substantial fraction of the tdTom was mislocalized to the apicoplast with some signal distributed in the parasite’s cytoplasm. By Western blot, vYFP was detected as both the isolated protein and the tdTom fusion (~ 100 kDa). Presumably, the fusion product accounted for the majority of the mislocalized tdTom, while the cytosolic fraction arose due to the expected T2A behaviour. These data suggest that ‘ribosome skipping’ might be less efficient and/or the downstream protein is more often misdirected when the upstream protein is apicoplast-targeted, at least within the context tested by the present constructs. This outcome is reminiscent of “slipstreaming” observed when using T2A for multi-cistronic expression of secreted proteins in mammalian cells, though this phenomenon is thought to be primarily influenced by the C-terminal portion of the upstream protein [36], which does not vary across the constructs used here. However, since the vYFP-2A-ATS-tdTom construct exhibited the expected subcellular targeting patterns, and given the other combinations in which proper subcellular trafficking was observed, some rules for using T2A to successfully achieve multi-cistronic protein expression with proper subcellular targeting have been defined.

## Discussion

A validated set of broadly useful plasmid vectors has been developed that enables versatile assembly of *P. falciparum* constructs, a frequently time-consuming process given the (A + T)-richness and large size of final vectors. This has been achieved by integrating simultaneous access to the high efficiency and inexpensively available yeast homologous recombination, Gibson assembly and conventional restriction/ligation cloning strategies. All three strategies are technically straightforward and use the same restriction enzyme-digested vector and PCR products generated with the same primer set. In principle, all three strategies can be executed in parallel or used interchangeably without the need for new genetic reagents, and can be used to successfully clone both reporter and native *P. falciparum* genes. Additionally, several widely used utility features for enhancing plasmid segregation and site-specific chromosomal integration have been pre-installed, and validated operations to enable user-tailored modifications to this vector family are provided.

In addition to improving the ease of constructing new *P. falciparum* expression vectors, a strategy for increasing the amount of expressible information that can be encoded on a single plasmid using a minimal set of 5′/3′-UTR cassettes was also developed. From a technical standpoint, this is especially useful as it simplifies the vector construction process by reducing overall plasmid size and instability during propagation in *E. coli*. Practically, this provides more efficient avenues for addressing questions in parasite biology requiring the co-expression of multiple genes. For example, several antigenically variant, multi-gene families, such as PfEMP1, STEVORs and RIFINs, are combinatorially expressed by the parasite to modulate host-parasite interactions, such as immune recognition and evasion [37,38]. Therefore, the ability to achieve pre-determined expression of specific combinations of these proteins could prove useful in understanding their combined contributions to these outcomes. Furthermore, subsets of proteins involved in multi-gene pathways must often be trafficked to distinct subcellular compartments. The tricarboxylic acid cycle [39,40], lipid and isoprenoid biosynthesis [41] and haem biosynthesis [42] involve multiple proteins distributed between the cytosol, mitochondrion and apicoplast, or exclusively targeted to one of these organelles. Also, a substantial fraction of the parasite-encoded proteome is trafficked to the host RBC, including the antigenically variant STEVOR and RIFIN families, and a significant number of these trafficked proteins play essential but poorly understood roles [43]. Therefore, strategies for achieving multi-cistronic expression while simultaneously preserving faithful protein trafficking will be broadly useful for studying parasite biology. Altogether, these openly available tools and validated methods should provide a convenient option for routinely generating *P. falciparum* plasmid vectors.

## Abbreviations

nptII: Neomycin phosphotransferase II; bsd: Blasticidin S deaminase; hdhfr: Human dihydrofolate reductase; ydhodh: Yeast dihydroorotate dehydrogenase.

## Competing interests

The authors declare they have no conflict of interests.

## Authors’ contributions

JCW, SJG and SMG designed DNA constructs, built plasmids, transfected parasites and performed western blots. JCW performed luciferase time course and qPCR experiments. SJG performed Northern blots. SMG and MCSL performed fluorescence microscopy and image processing. All authors analysed data. JCW, SJG, SMG and JCN wrote the manuscript, with input from MCSL and DAF. JCN supervised the project. All authors read and approved the final manuscript.

## Supplementary Material

Additional file 1 Oligonucleotides used in vector construction. Click here for file

Additional file 2 List of primers used for quantitative PCR. Click here for file

Additional file 3 Vectors used in this study. Click here for file
