# Supplementary material for: An integrated strategy for efficient vector construction and multi-gene expression in Plasmodium falciparum
Source: Malar J. 2013 Oct 26;12:373. doi: 10.1186/1475-2875-12-373 (PMC3842810; doi:10.1186/1475-2875-12-373)
Supplement: Additional file 1 — Oligonucleotides used in vector construction. [file 1475-2875-12-373-S1.pdf]

Additional file 2. Oligonucleotides used in vector construction.

Primer	Sequence	Notes
SG311	GAAATATATCAGACGTCTCCCCCGGACCATGGAAGACGCCAAAAACATAAAGAAAGGCC	*FL* probe (forward)
SG313	GACCCCATTGTGAGTACATAAATATATTATATAACTCGAGTTACAACTCGGACTTTCCGC	*FL* probe (reverse)
SG763	AGCATGTGCATGGCATCCCCTT	Amplifying uniquely identifying regions of vYFP-tdTom cassette (forward)
SG764	TGACCTCCTCGCCCTTGCTCA	Amplifying uniquely identifying regions of vYFP-tdTom cassette (reverse)
SG502	AGTAGCATCACCTTCACCTTCACC	Sequencing from vYFP in dicistronic vYFP-tdTom constructs (reverse)
SG646	CTGCCTTATCCAAAGATCCAAACG	Sequencing from vYFP in dicistronic vYFP-tdTom constructs (forward)
SG702	TAGGTGACACTATAGAATACTCAAGCTTGGCGGCCGCCCGAGCTCGAATTCCGGGTTTGT	Tandem *attP* (forward)
SG703	AGTTAATTCATCAAATAGCATGCCTGCAGGTCGACGCCAGGGTTTTCCCAGTCACGA	Tandem *attP* (reverse)
SG814	GGACATTGTTTAAACGAGCAGG ACGCGT TGAATTGTCCCCACGCCGCGCCC	Recircularizion at *PmlI*/*MluI* sites (forward)
SG815	CCTGCTCGTTTAAACAATGTCC CACGTG ATGAAAAGGACCCAGGTGGCA	Recircularizion at *PmlI*/*MluI* sites (reverse)
SG864	GCAGGTCGACGCCAGGGTTT	Confirming pfYC integration at *cg6-attB* (forward)
SG865	GACGCCGGGCAAGAGCAACT	Confirming pfYC integration at *cg6-attB* (reverse)
SG928	GGGCGCGGCGTGGGGACAATTCAACGCGTTAATTATTAATATATTAATTATTTAGACTTA	*pfcen5-1.5* PCR amplification (forward)

SG894	TGCCACCTGGGTCCTTTTCATCACGTG TATGTATAATTAAATTAAATATTATAAACA CAC	*pfcen5-1.5* PCR amplification (reverse)
SG369	CCAATGCTTAATCAGTGAGGC	Sequencing primer: yCEN/or *pfcen5-1.5* region
Trx pcDT F	AAATATATACACACACCTAAAACTTACAA AGTATCCTAGGAAAAATGAACAATGTAAT TT	*trxR* PCR amplification (forward)
Trx pcDT R	TTTAATCTATTATTAAATAAATTTAATGGG GTACCCGCGGTTATCCACATTTTCCACCCC	*trxR* PCR amplification (reverse)
AMA1 pcDT F	AAATATATACACACACCTAAAACTTACAA AGTATCCTAGGAAAAATGAGAAAATTATA CT	*ama1* PCR amplification (forward)
AMA1 pcDT R	TTTAATCTATTATTAAATAAATTTAATGGG GTACCCGCGGTTAATAGTATGGTTTTTCCA	*ama1* PCR amplification (reverse)
