# Supplementary material for: An integrated strategy for efficient vector construction and multi-gene expression in Plasmodium falciparum
Source: Malar J. 2013 Oct 26;12:373. doi: 10.1186/1475-2875-12-373 (PMC3842810; doi:10.1186/1475-2875-12-373)
Supplement: Additional file 2 — List of primers used for quantitative PCR. [file 1475-2875-12-373-S2.pdf]

Additional file 3. List of primers used for quantitative PCR.

**Primer**	**Sequence**	**Target gene**	**Source**
Neo (forward)	CATCCTGATCGACAAGACCG	nptII	This work
Neo (reverse)	CCTGCCGAGAAAGTATCCATC	nptII	This work
BSD (forward)	GCTGTCCATCACTGTCCTTC	Blasticidin S deaminase	This work
BSD (reverse)	TGGCAACCTGACTTGTATCG	Blasticidin S deaminase	This work
hDHFR (forward)	GAGGTTGTGGTCATTCTCTGG	Human dihydrofolate reductase	This work
hDHFR (reverse)	AGAACATGGGCATCGGC	Human dihydrofolate reductase	This work
yDHODH (forward)	TCCACCTGTACCGATAACTTTG	Yeast dihydroorotate dehydrogenase	This work
yDHODH (reverse)	GATGTGGAGAAGGAGAGTGTAG	Yeast dihydroorotate dehydrogenase	This work
Pf-ß-actin (forward)	AAAGAAGCAAGCAGGAATCCA	*P. falciparum* ß-actin (PF3D7_1246200)	(1)
Pf-ß-actin (reverse)	TGATGGTGCAAGGGTTGTAA	*P. falciparum* ß-actin (PF3D7_1246200)	(1)

Reference

1. Augagneur, Y., Wesolowski, D., Tae, H.S., Altman, S. and Ben Mamoun, C. (2012) Gene selective mRNA cleavage inhibits the development of *Plasmodium falciparum*. *Proc Natl Acad Sci USA*, **109**, 6235-6240.
