# Supplementary material for: An integrated strategy for efficient vector construction and multi-gene expression in Plasmodium falciparum
Source: Malar J. 2013 Oct 26;12:373. doi: 10.1186/1475-2875-12-373 (PMC3842810; doi:10.1186/1475-2875-12-373)
Supplement: Additional file 3 — Vectors used in this study. [file 1475-2875-12-373-S3.pdf]

Additional file 1. Vectors used in this study

Vector	Selection in *P. falciparum*	Assembly method	GenBank accession	Notes
pfYC101:FL	G-418	Yeast HR		Rep20, yeast centromere; FL and resistance marker are in cassettes A and B, respectively
pfYC110:FL	G-418	Yeast HR		Rep20, yeast centromere; Resistance marker and FL are in cassettes A and B, respectively
pfYC102:FL	Blasticidin S	Yeast HR		Rep20, yeast centromere; FL and resistance marker in cassettes A and B, respectively
pfYC120:FL	Blasticidin S	Yeast HR		Rep20, yeast centromere; Resistance marker and FL are in cassettes A and B, respectively
pfYC120:RL	Blasticidin S	Yeast HR		Rep20, yeast centromere; Resistance marker and FL are in cassettes A and B, respectively; Used in double transfection experiment (Figure 4B)
pfYC103:FL	WR99210	Yeast HR		Rep20, yeast centromere; FL and resistance marker are in cassettes A and B, respectively
pfYC130:FL	WR99210	Yeast HR		Rep20, yeast centromere; Resistance marker and FL are in cassettes A and B, respectively
pfYC104:FL	DSM-1	Gibson		Rep20, yeast centromere; FL and resistance marker are in cassettes A and B, respectively

pfYC140:FL	DSM-1	Gibson		Rep20, yeast centromere; Resistance marker and FL are in cassettes A and B, respectively
pfYC320:FL	Blasticidin S	Gibson		Contains 2xattP element; No Rep20 or yeast centromere elements; Resistance marker and FL are in cassettes A and B, respectively
pfYC340:FL	DSM-1	Gibson		Contains 2xattP element; No Rep20 or yeast centromere elements; Resistance marker and FL are in cassettes A and B, respectively
pfYC120:vYFP-2A-tdTom	Blasticidin S	Gibson		vYFP and tdTom expression in the parasite cytosol
pfYC120:vYFP-2A-(ATS)-tdTom	Blasticidin S	Gibson		vYFP expression in the parasite cytosol and tdTom targeting to the apicoplast
pfYC120:vYFP-2A-(MTS)-tdTom	Blasticidin S	Gibson		vYFP expression in the parasite cytosol and tdTom targeting to the mitochondrion
pfYC120:vYFP-2A-(PEX)-tdTom	Blasticidin S	Gibson		vYFP expression in the parasite cytosol and tdTom export to the RBC cytosol
pfYC120:(MTS)-vYFP-2A-(MTS)-tdTom	Blasticidin S	Gibson		vYFP and tdTom targeting to the mitochondrion
pfYC120:(PEX)-vYFP-2A-(PEX)-tdTom	Blasticidin S	Gibson		vYFP and tdTom export to the RBC cytosol
pfYC120:(ATS)-vYFP-2A-tdTom	Blasticidin S	Gibson		vYFP targeted to the apicoplast and tdTom to the parasite cytosol
